# Exploration and practice of AI-enabled smart caregiver-free ward with traditional Chinese medicine characteristics: a case study based on the Guangming branch of Shenzhen Traditional Chinese Medicine Hospital

**DOI:** 10.3389/fpubh.2026.1799247

**Published:** 2026-06-02

**Authors:** Siping Peng, Yuan Gao, Yu Hong, Xiaoyan Ren, Xiongwei Tang, Xiaoying Ding, Jingjing Li, Jing Wang, Daijiong Guo

**Affiliations:** 1 The Fourth Clinical Medical College, Guangzhou University of Chinese Medicine, Shenzhen, China; 2 Department of Endocrinology, Shenzhen Traditional Chinese Medicine Hospital, Shenzhen, China

**Keywords:** artificial intelligence, caregiver-free ward, digital therapeutics, hospital management, smart nursing, smart ward

## Abstract

**Background and purpose:**

Integrating artificial intelligence (AI), with Traditional Chinese Medicine (TCM) principles remains a key challenge for digitalized hospital advancement. This study aims to systematically elaborate on the practical pathway, technological architecture, and preliminary outcomes of establishing smart TCM-characterized, caregiver-free wards at the Guangming New Campus of Shenzhen Traditional Chinese Medicine Hospital, thereby providing an exemplar for the industry.

**Methods:**

We adopted a two-pronged strategy encompassing top-level design and phased implementation. Centered on the localized deployment of a domestic AI large language model (DeepSeek), we constructed an open-architecture smart ward platform that integrates Internet of Things (IoT), big data analytics, and 5G technology. This platform comprises six core modules: intelligent admission-discharge-transfer, TCM-informed smart education, patient safety monitoring, smart logistics, continuous care, and intelligent operation maintenance. Crucially, these modules are deeply embedded with distinctive TCM elements, including the midnight-noon ebb-flow doctrine, five-element music therapy, and constitution-based health preservation.

**Results:**

Following the implementation, significant improvements were observed across key metrics. Patient satisfaction with the healthcare experience rose to 98.53%. Health literacy regarding TCM among patients increased from a baseline of 70% to 92.00%. The bedside discharge settlement rate reached 99.2%, accompanied by a notable reduction in associated healthcare costs. AI-enabled automation reduced direct caregiver tasks including vital signs collection (saving 20.8 min/patient/day), documentation (saving 28.7 min/patient/day), and patient education, contributing to the feasibility of the caregiver-free model. The paperless inpatient ward utilizes smart terminals and IoT technologies to achieve fully digitalized management of medical orders, nursing, medication, and testing, thereby reducing paper waste, lowering costs, and improving healthcare quality, efficiency, and safety.

**Conclusion:**

This study demonstrates that the establishment of an AI-enabled, TCM-characterized smart caregiver-free ward effectively enhances healthcare quality, patient experience, and operational efficiency. It represents a successful and innovative fusion of traditional TCM wisdom with modern information technology, providing a solid empirical foundation for the modernization and service model transformation of TCM hospitals.

## 1 Introduction

The global healthcare system is grappling with multifaceted challenges, including resource maldistribution, escalating costs, and workforce shortages, exacerbated by an aging population and the growing burden of chronic diseases. Within this context, the social dilemma of “one hospitalization, exhausting the whole family” has become a widespread societal pain point. In response, China has been actively promoting the “caregiver-free” ward model. Its core principle is to replace family members with certified healthcare assistants, who assume responsibility for both daily living assistance and professional nursing care during hospitalization (1).

Concurrently, the burgeoning advancement of artificial intelligence (AI) technology is forging novel pathways for the digital transformation of the healthcare sector. The “Implementation Opinions on Promoting and Regulating the Application Development of ‘AI + Healthcare”', jointly issued in 2025 by China's National Health Commission (NHC) and multiple other ministries, explicitly outlines the imperative to deepen the integration of AI across clinical diagnosis and treatment, patient services, and Traditional Chinese Medicine (TCM). This policy provides crucial support and practical guidance for the development of intelligent, caregiver-free wards.

The application of AI in TCM has evolved from initial technical explorations to a more systematic and structured paradigm. Technically, advanced algorithms, such as multi-task meta-attention networks and TCM-specialized large language models, are now deployed for critical tasks including tongue and pulse image analysis, syndrome element recommendation, and herbal formula generation. These advancements have significantly enhanced the objectivity of syndrome differentiation and the precision of prescription formulation. In terms of application scenarios, AI now covers the entire patient journey—from pre-consultation and diagnosis to post-treatment care—encompassing intelligent pre-consultation, AI-assisted clinical decision-making, and automated rehabilitation medical record generation (2). These advancements have paved the technical way for the systematic application of AI in TCM-characterized caregiver-free wards.

As a unique healthcare resource in China, Traditional Chinese Medicine possesses distinct advantages in preventive care (“treating the pre-disease”), rehabilitation, and holistic regulation. However, its traditional diagnostic and treatment model is inherently reliant on empirical knowledge, posing significant challenges to the standardization and scalability of individualized syndrome differentiation. This, to some extent, constrains the accessibility and consistency of TCM services. Consequently, a pivotal challenge for the current inheritance and development of TCM is how to synergistically integrate AI with its unique characteristics to construct a smart service model that “upholds its orthodox principles while driving innovation.”

The development of smart hospitals has emerged as a central engine driving the high-quality development of the healthcare service system (3). National policies explicitly mandate “leveraging information technology to optimize nursing service processes and models” and “promoting the development of TCM nursing,” with particular emphasis on extending TCM nursing services into community and home-based settings (4).

The integration of AI and robotics into critical care environments has emerged as a transformative force in global healthcare delivery, particularly within intensive care units (ICUs) and specialized ward settings. A growing body of international evidence demonstrates that AI technologies—including machine learning (ML), natural language processing (NLP), and predictive analytics—are fundamentally reshaping clinical decision-making, risk assessment, and patient monitoring paradigms (5, 6). These innovations have progressed from theoretical frameworks to practical implementations, with real-time monitoring systems now capable of continuous physiological data acquisition (heart rate, blood pressure, respiratory rate, oxygen saturation) that enables early identification of clinical deterioration and facilitates timely therapeutic interventions (7).

In high-income countries (HICs), remote patient monitoring (RPM) systems have matured into integral components of chronic disease management, with robust evidence supporting their clinical utility and economic (8). A 2022 systematic review of economic evaluations confirmed that RPM is highly cost-effective for managing hypertension and heart failure, resulting in reduced hospital readmissions and improved therapeutic adherence (9). A large United States cohort study (8) involving 6,595 hypertensive patients reported a mean systolic blood pressure reduction of 7.3 mmHg following long-term RPM participation, while a United Kingdom meta-analysis demonstrated that telemonitoring achieved a 3.7 mmHg greater reduction in systolic blood pressure compared with usual care.

Parallel advancements have occurred in fall detection and prevention systems for institutional settings. A systematic review of digital healthcare approaches across hospitals and long-term care facilities evaluated 33 studies comprising 20 fall detection systems and 13 fall prediction models (10). These systems utilize inertial sensors, pressure sensors, radar, and multimodal sensing technologies to achieve high usability rates exceeding 80% acceptance among users. Prediction models incorporating gradient boosting and neural networks have demonstrated moderate-to-strong discriminatory power for continuous gait features, while regression and boosting approaches have proven effective for analyzing categorical electronic health record data. However, despite improved surveillance capabilities, these detection systems have not consistently reduced fall incidence or injurious falls—one trial reported a non-significant reduction, while another observed a non-significant increase, with frequent false alarms contributing to clinician alarm fatigue (11).

The evolution toward “smart hospitals” and Healthcare 4.0 has further accelerated internationally, with multilayered design frameworks encompassing sensing, networking, remote services, knowledge generation, and application layers (12). Key enabling technologies—including the Internet of Things (IoT), cloud computing, extended reality, big data analytics, Building Information Modeling (BIM), digital twins, and ML algorithms—have shaped these architectural innovations across developed countries. Concurrently, AI-driven RPM systems integrating IoT devices and ML algorithms have demonstrated effectiveness in early disease detection, personalized care delivery, and reduction of hospital readmissions in home-based settings (13, 14).

Despite these technological advances, significant limitations persist in the international literature. Most prediction models lack external validation and remain disconnected from clinical care pathways. Frequent false alarms contribute to alert fatigue among clinicians, undermining the clinical utility of these systems. Furthermore, traditional automation approaches struggle to adapt to dynamic and complex clinical environments, frequently requiring manual programming without autonomous learning or decision-making capabilities. Critically, while human-centered design and sustainability have been highlighted as essential principles, the integration of holistic medical philosophies—such as those embodied by Traditional Chinese Medicine (TCM)—remains conspicuously absent from international smart ward frameworks (15, 16).

Against this backdrop, a critical challenge has surfaced: how to harness a new generation of information technologies—including AI, the Internet of Things (IoT) (17), and big data—to empower TCM diagnosis, treatment, and nursing practice. The ultimate goal is to construct a smart healthcare system that embodies both the conveniences of modern technology and the rich cultural essence of TCM. whereas existing smart ward implementations in HICs operate within resource-rich contexts with robust infrastructure, our study demonstrates feasibility in a real-world clinical setting that balances technological innovation with culturally specific health philosophies. This provides a replicable model for integrating traditional medical systems with AI-enabled care delivery—an area identified as critically underexplored in the literature.

Shenzhen Traditional Chinese Medicine Hospital, as one of China's first Tertiary Grade A TCM hospitals and a key TCM hospital under the National TCM Inheritance and Innovation Project, has consistently been at the forefront of TCM informatization. It has achieved Level 6 in the Electronic Medical Record (EMR) System Function Application Assessment and Level 5B in the Standardized Maturity Evaluation for Interoperability, establishing a robust foundation for its intelligent initiatives (18). In March 2025, the hospital successfully completed the localized deployment of DeepSeek, a domestically developed large AI model, positioning itself as a pioneer in integrating AI with TCM diagnosis and treatment nationwide. Its new branch, the Shenzhen TCM Hospital of Guangming Branch, now the largest single-structure TCM hospital project in China, is guided by a development strategy integrating “clinical care, education, research, prevention, health preservation, rehabilitation, and translation.” This branch has embarked on cutting-edge exploration in this field, deeply integrating AI with TCM characteristics to create a “caregiver-free” smart ward model. This initiative aims to transform traditional care into intelligent, precise, and human-centric services.

Capitalizing on this foundation, the Guangming Branch of Shenzhen TCM Hospital selected key departments, such as the Soft Tissue Injury Department, as pilot units to orchestrate a systematic initiative in exploring and practicing TCM-characterized smart wards. This effort extends AI capabilities across the entire inpatient service chain. Through the deployment of a medical domain-specific large language model, the development of an intelligent TCM syndrome differentiation system, and the implementation of bedside intelligent terminals coupled with a contactless vital signs monitoring network, the hospital has achieved refined patient care, real-time risk report, and personalized rehabilitation interventions. This integrated approach has culminated in the preliminary establishment of a prototype “Smart Caregiver-Free” ward model. Unlike previous fall detection and monitoring systems that have shown limited preventive effects despite improved surveillance, our approach embeds predictive algorithms within a patient-centered workflow guided by TCM's holistic principles, potentially addressing the “prevention paradox” identified in international studies. This case study, centered on the Guangming Branch, systematically investigates how AI empowers the construction and operation of TCM-characterized smart caregiver-free wards. The analysis will be conducted across multiple dimensions, including service model re-engineering, technology architecture integration, and pathways for TCM-Western medicine fusion. It will evaluate the model's potential value in enhancing nursing quality, optimizing operational efficiency, and improving the patient experience. Through this case study, we aim to provide transferable empirical insights and a theoretical framework for the global modernization of TCM, the deep application of AI in healthcare, and the innovation of high-quality, humanized medical care delivery models.

## 2 Methods

### 2.1 Top-level design and organizational structure

At the project's inception, the hospital established a dedicated “Guangming Campus Launch Task Force”, directly overseen by the hospital executive leadership, to orchestrate a multi-departmental collaborative mechanism. The team strictly adhered to the core principles of being “patient-centered, TCM-featured, and data-driven,” and adopted an implementation strategy of “phased rollout, pilot-first, and scaling upon maturation” to ensure the project's scientific and orderly progression.

**Figure 1:**
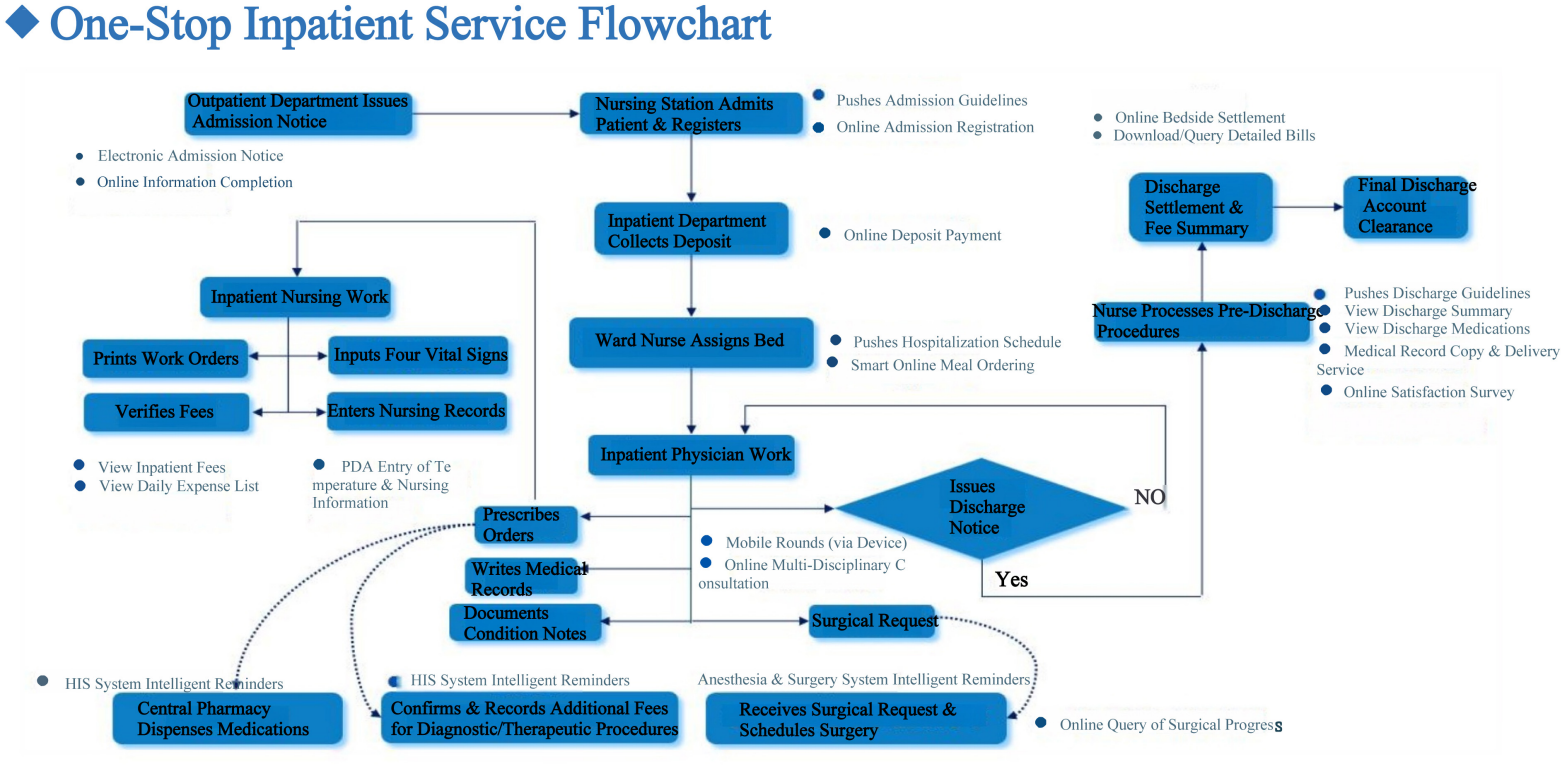
One-stop inpatient service flowchart.

We have established an intelligent one-stop admission and discharge processing system, which encompasses a fully paperless service throughout the entire patient journey. This includes bedside admission procedures, in-hospital examinations and treatments, completion of all required formalities at the bedside, and discharge services, as depicted in Figure 1.

### 2.2 Technical platform architecture

**Figure 2:**
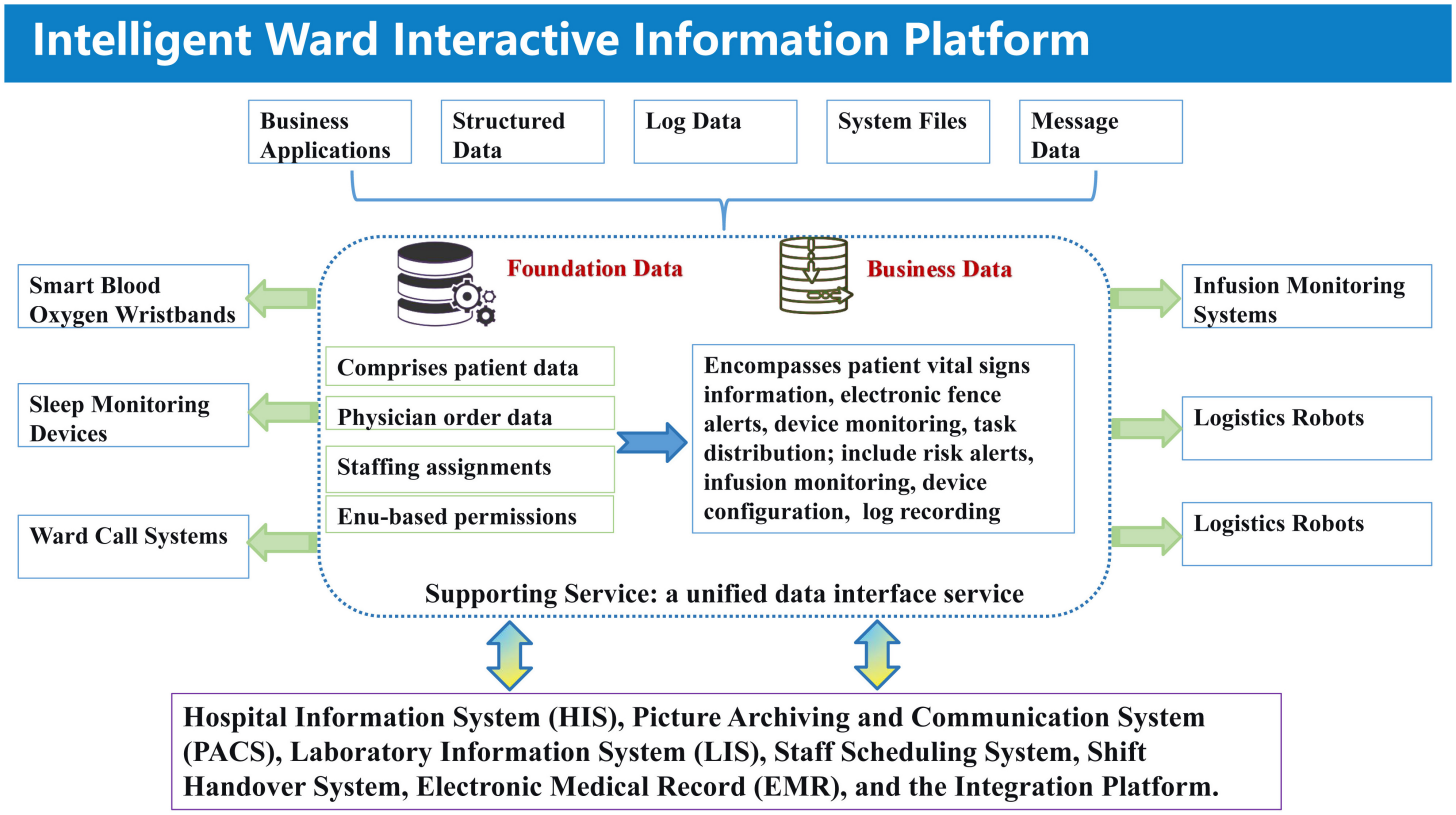
Intelligent ward interactive information platform.

As Figure 2 shows that, We constructed a layered, decoupled, and openly integrated smart ward interactive information platform as the core technological foundation.

- Infrastructure layer: this layer leverages a 10-gigabit Ethernet backbone, comprehensive 5G coverage, and a hybrid cloud architecture to ensure high-speed data transmission and robust computational capabilities.
- Data middle platform: this platform integrates the Hospital Information Platform (HIP) to establish a unified master patient index (MPI). It consolidates data from diverse systems, including the hospital information system (HIS), laboratory information system (LIS), picture archiving and communication system (PACS), electronic medical record (EMR), as well as intelligent shift-handover and scheduling systems, forming a standardized and structured data center.
- AI capability layer: the locally deployed DeepSeek large language model provides core AI functionalities, such as natural language processing (NLP), knowledge graph construction, and predictive analytics.
- Application support layer: an enterprise service bus (ESB) is employed to achieve interoperability between systems. This layer supports the “plug-and-play” integration and unified data ingestion from various Internet of Things (IoT) devices, such as smart wristbands, vital signs monitors, and augmented reality (AR) equipment.
- Business application layer: a suite of smart applications, encompassing patient services, clinical nursing, and operational management, has been developed on top of this platform.

### 2.3 System architecture and interoperability framework

The proposed smart ward platform adopts a five-layer horizontal architecture with three vertical cross-cutting layers, following established IoT healthcare architectural patterns (18). This design ensures clear separation of concerns, standardized data exchange, and seamless integration with existing hospital information systems.

#### 2.3.1 Horizontal architecture layers

##### Layer 1: Perception layer (Data acquisition)

The perception layer comprises all data sources, including:

- Wearable devices: continuous vital sign monitors (heart rate, blood pressure, oxygen saturation) transmitting via Bluetooth Low Energy (BLE)
- Smart bed sensors: pressure distribution, patient movement, and sleep quality monitors
- Medical devices: connected infusion pumps, ventilators, and bedside monitors
- Clinical information systems: hospital information system (HIS), laboratory information system (LIS), and picture archiving and communication system (PACS)

##### Layer 2: Network and IoT gateway layer

This layer provides edge computing capabilities and protocol translation:

- Edge processing nodes deployed at ward level perform:
  Data filtering (removing artifacts and outliers)
  Format normalization (converting proprietary formats to standard schemas)
  Real-time alerts for critical values (reducing latency by 70% compared to cloud-only processing)
- Protocol translation: MQTT/CoAP to HL7 FHIR, proprietary DICOM to DICOMweb

##### Layer 3: Data bus and API gateway layer

A unified Enterprise Service Bus (ESB) with API Gateway manages all system integration:

- Message routing: Apache Kafka for high-throughput event streaming
- Protocol mediation: bidirectional translation between HL7 v2, FHIR, and DICOM
- Security enforcement: OAuth 2.0 authentication, TLS 1.3 encryption, ATNA audit logging
- Service discovery: dynamic registration of microservices

##### Layer 4: Knowledge layer (AI capability layer)

This layer provides intelligent processing and decision support, addressing the reviewer's concern about ambiguous AI functionality. It comprises five specialized AI modules.

Key innovation: the Knowledge Layer operates on an event-driven architecture. Each module subscribes to relevant Kafka topics (e.g., patient.vitals.updated, lab.result.available) and publishes results asynchronously, ensuring loose coupling and scalability.

##### Layer 5: Application layer

End-user applications for different stakeholders:

- Clinical dashboard (React.js): real-time patient monitoring, alert management
- Nurse mobile app (React Native): task assignment, vital sign confirmation
- Patient portal (iOS/Android): education content, survey completion
- Management console: analytics, quality metrics, operational dashboards

#### 2.3.2 Vertical cross-cutting layers

##### Security and privacy layer

- Authentication: OAuth 2.0 with OpenID Connect (Keycloak)
- Authorization: role-based access control (RBAC) with FHIR security labels
- Audit: IHE ATNA profile compliant logging
- Data protection: encryption at rest (AES-256) and in transit (TLS 1.3)

##### Management and orchestration layer

- Container orchestration: Kubernetes for microservices deployment
- Service mesh: Istio for traffic management, observability
- Configuration management: centralized with GitOps

##### Integration and interoperability layer

- Standards compliance: HL7 FHIR R4 (core resources), DICOM, IHE XDS.b
- Terminology services: SNOMED CT, LOINC, ICD-11, TCM terminology standards (GB/T 15657-2021)
- Legacy system adapters: HL7 v2 MLLP gateways, DICOM converters

#### 2.3.3 Educational robot mechanism

The patient education robot employs a Retrieval-Augmented Generation (RAG) architecture:

(1) Knowledge base construction:
  - TCM health preservation literature (ancient texts, modern guidelines)
  - Hospital-specific patient education materials
  - Frequently asked questions database
  - All content vectorized and stored in Pinecone vector database
(2) Personalization engine:
  - Patient profile (age, diagnosis, literacy level, language preference) from FHIR Patient resource
  - Current treatment context from FHIR Encounter and CarePlan
  - Learning style inferred from interaction history
(3) LLM integration:
  - Deploys Baichuan2-7B (privatized for data privacy) or optional GPT-4 API for enhanced capabilities
  - Prompt engineering with RAG-retrieved context ensures accurate, personalized responses
  - Response generated in patient's preferred language and reading level
(4) Delivery channels:
  - Bedside tablet interface (text + multimedia)
  - Mobile app push notifications
  - Voice-enabled interaction via smart speaker
(5) Clinical Integration:
  - Education completion recorded in FHIR Communication resource
  - Patient comprehension assessed via follow-up questions
  - Alerts to nursing staff if patient requires additional explanation

**Figure 3:**
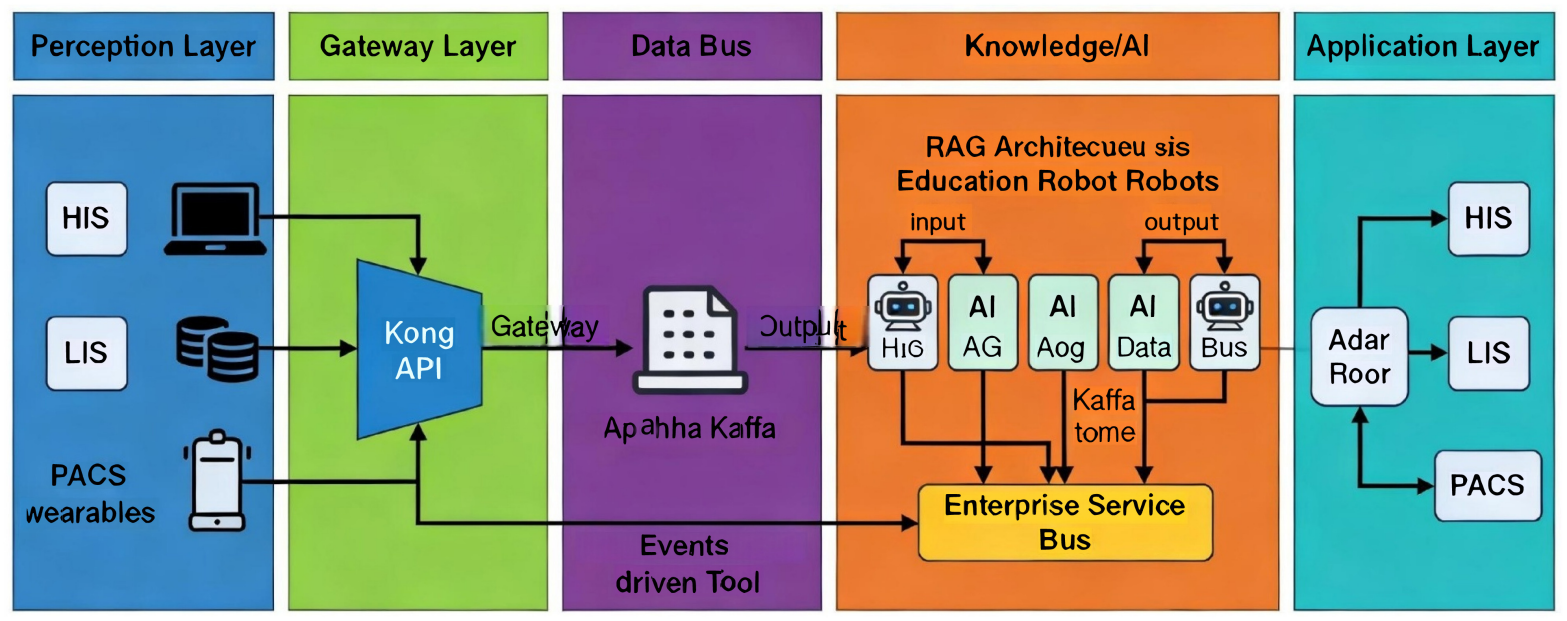
UML component deployment diagram.

As Figure 3 and Supplementary Table 3 (Supplementary Materials) show, we have completely redesigned Figure 3 as a comprehensive UML deployment diagram (C4 model container view) showing:

- All five horizontal layers with component-level detail
- API Gateway and Enterprise Service Bus explicitly shown
- Data flows with labeled interfaces (protocols, data formats)
- Integration points with HIS/LIS/PACS/wearables
- AI Knowledge Layer modules with their inputs/outputs

The diagram illustrates the five-layer horizontal architecture with vertical cross-cutting concerns. Components are color-coded by layer: Perception (blue), Gateway (green), Data Bus (purple), Knowledge/AI (orange), and Application (teal). Data flows are annotated with interface protocols (HL7 FHIR, MQTT, gRPC) and data formats. The API Gateway (Kong) manages all external access, while Apache Kafka provides event-driven communication between microservices. Integration with legacy hospital systems (HIS, LIS, PACS) occurs through dedicated adapters that translate between legacy protocols and FHIR R4. The AI Knowledge Layer comprises five specialized modules, each subscribing to relevant Kafka topics and publishing results asynchronously. Educational robot functionality is implemented via a RAG architecture with privatized LLM deployment.

### 2.4 Development of core modules with deep TCM integration

Leveraging the aforementioned platform, we prioritized the development of the following core modules, each deeply integrated with TCM characteristics.

#### 2.4.1 Intelligent one-stop service and fully paperless process

- Bedside intelligent terminals: patients can complete the entire paperless admission process—from receiving the electronic admission certificate and information registration to insurance verification and online payment—at their bedside using integrated terminals or personal mobile devices, with the procedure averaging 5 min. Similarly, bedside discharge settlement can be completed in an average of 30 s.
- Process re-engineering: the inpatient service flow has been fundamentally re-engineered through the deployment of guidance robots, intelligent admission service platform SMS reminders, and bedside QR code payment options. This restructuring has significantly reduced unnecessary patient movement and physical transitions between departments.
- The paperless inpatient ward achieves fully digitalized management of the entire process—including medical orders, nursing care, medication administration, and laboratory testing—through the adoption of smart terminals, the Internet of Things (IoT), and other advanced technologies. This not only reduces the waste of resources such as paper, contributing to environmental sustainability, but also significantly lowers medical costs while enhancing the quality, efficiency, and safety of healthcare delivery.

#### 2.4.2 TCM-characterized smart education and rehabilitation system

This represents the core innovation of the project, aiming to materialize and personalize the TCM concepts of “preventive treatment of disease” and “syndrome differentiation-based nursing”.

**Figure 4:**
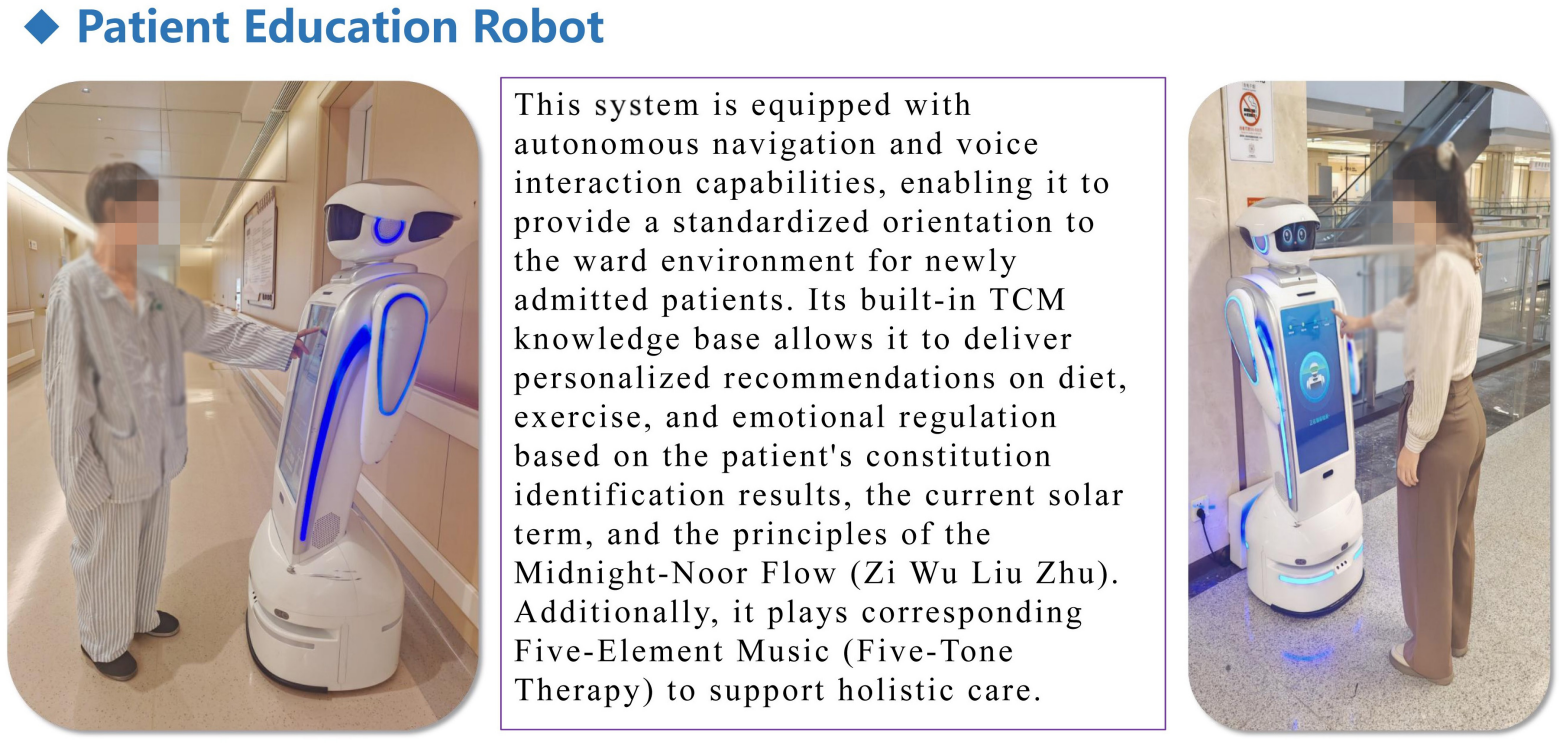
Patient education robot.

**Figure 5:**
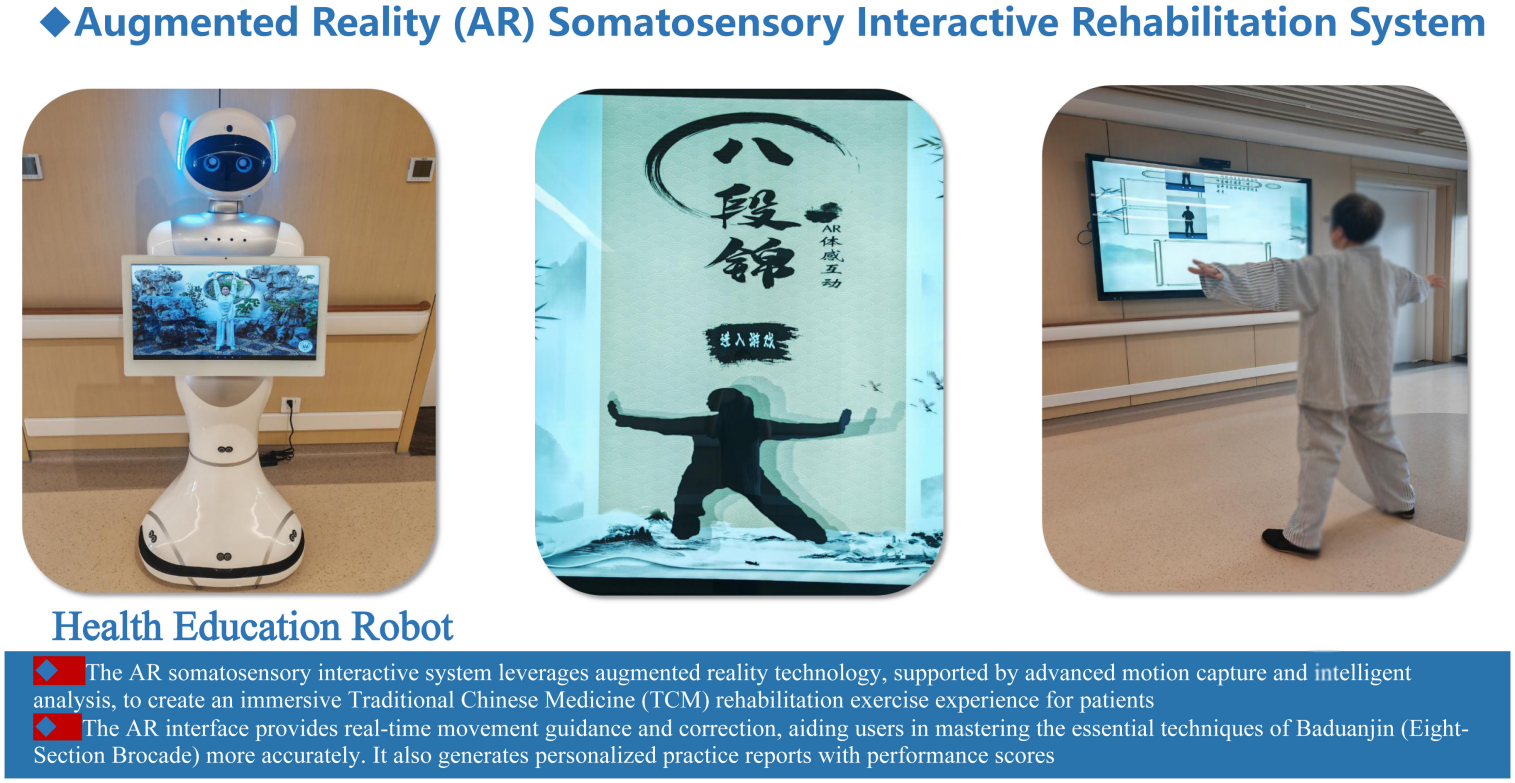
Augmented reality (AR) somatosensory interactive rehabilitation system.

- Education robot (Figure 4): equipped with autonomous navigation and voice interaction capabilities, the robot provides standardized orientation to the ward environment for newly admitted patients. Its built-in TCM knowledge base enables it to deliver precise, personalized recommendations on diet, exercise, and emotional regulation based on the patient's constitution identification results, the current solar term, and the principles of the midnight-noon ebb-flow theory (Ziwu Liuzhu). Furthermore, it plays corresponding Five-Element Music (Wuyin Liaofa) to support holistic care.
- AR somatosensory interactive rehabilitation system (Figure 5): this system utilizes AR and motion capture technologies to provide patients with immersive training in traditional health-preserving exercises such as Baduanjin (Eight-Section Brocade) and Wuqinxi (Five-Animal Frolics). The system captures patients' movement trajectories in real-time, compares them against standardized models, provides real-time visual feedback and guidance through the AR interface, and generates personalized exercise reports with performance scores.
- Bedside intelligent terminal health guidance: the system delivers structured TCM-based health preservation and rehabilitation content through bedside terminals. This includes, but is not limited to: the midnight-noon ebb-flow doctrine and twelve 2-h time period health preservation methods, meridian and acupoint patting exercises, Master Wei Guikang's cervical-lumbar exercises (inheriting the academic legacy of National TCM Master Wei Guikang), kidney-strengthening and body-invigorating ear massage techniques, and kidney-benefiting and brain-enhancing finger exercises. This comprehensive content addresses the rehabilitation needs of patients across different disease stages.
- Intelligent nursing plus management platform: serving as the “digital brain” for health education management, this platform conducts trend analysis, departmental ranking, and data drilling of key performance indicators—including the volume of health education push notifications, read rates, and patient engagement metrics—across the entire hospital. This enables quantitative evaluation and continuous quality improvement of health education initiatives.

#### 2.4.3 “Seamlessly connected and intelligently responsive” patient safety system

**Figure 6:**
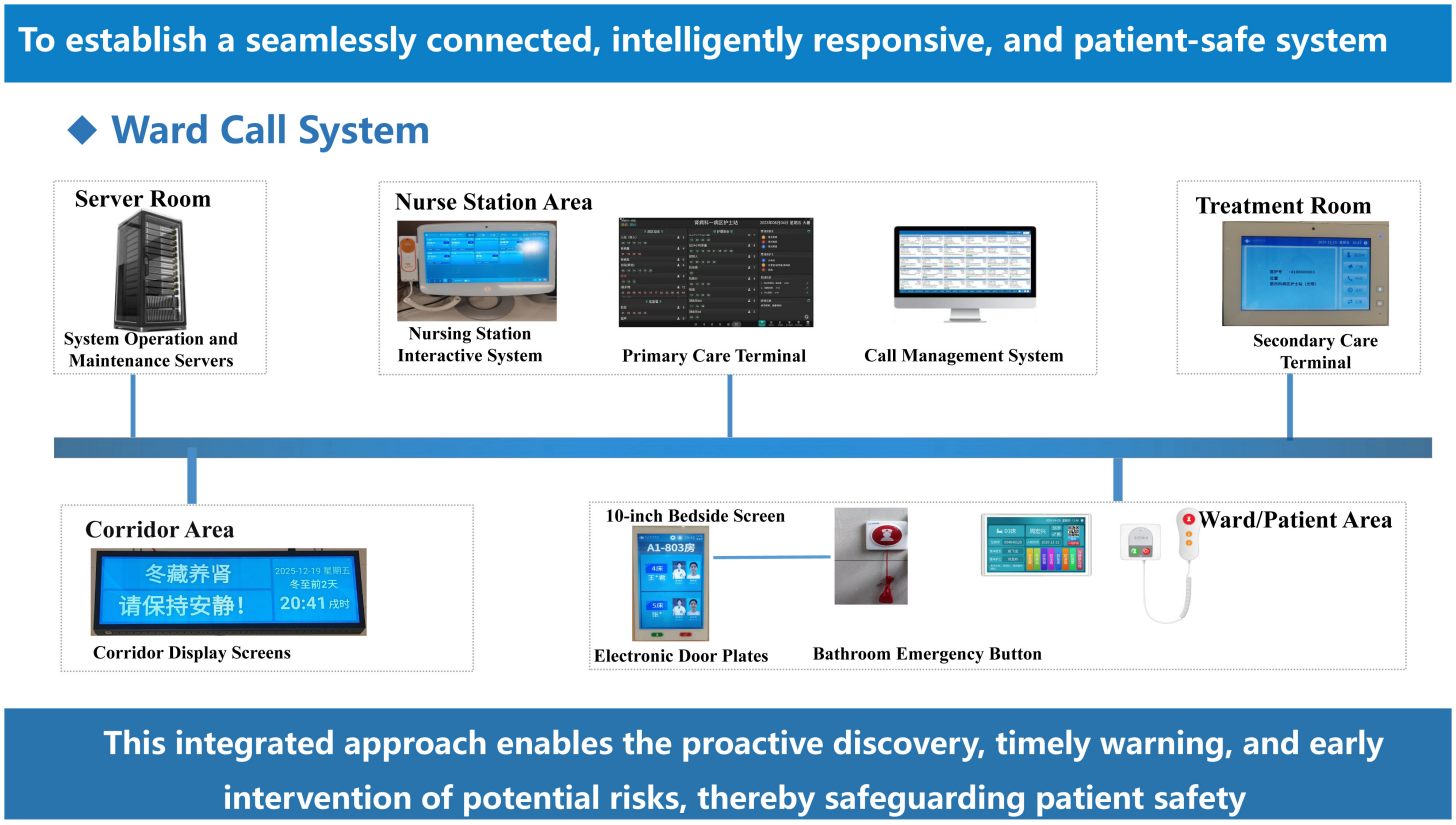
To establish a seamlessly connected, intelligently responsive, and patient-safe system.

Intelligent vital signs monitoring (Figure 6): the system automatically collects patients' vital signs data through intelligent vital signs monitors (measuring blood pressure, pulse, body temperature, and blood oxygen saturation) and pneumatic photoplethysmography (PPG) health monitoring wristbands. This data is transmitted to the HIS within 40 s via a single-click upload mechanism, completely replacing traditional manual recording and data entry methods. Additionally, sleep monitoring devices provide unobtrusive monitoring of sleep quality and related physiological parameters.

**Figure 7:**
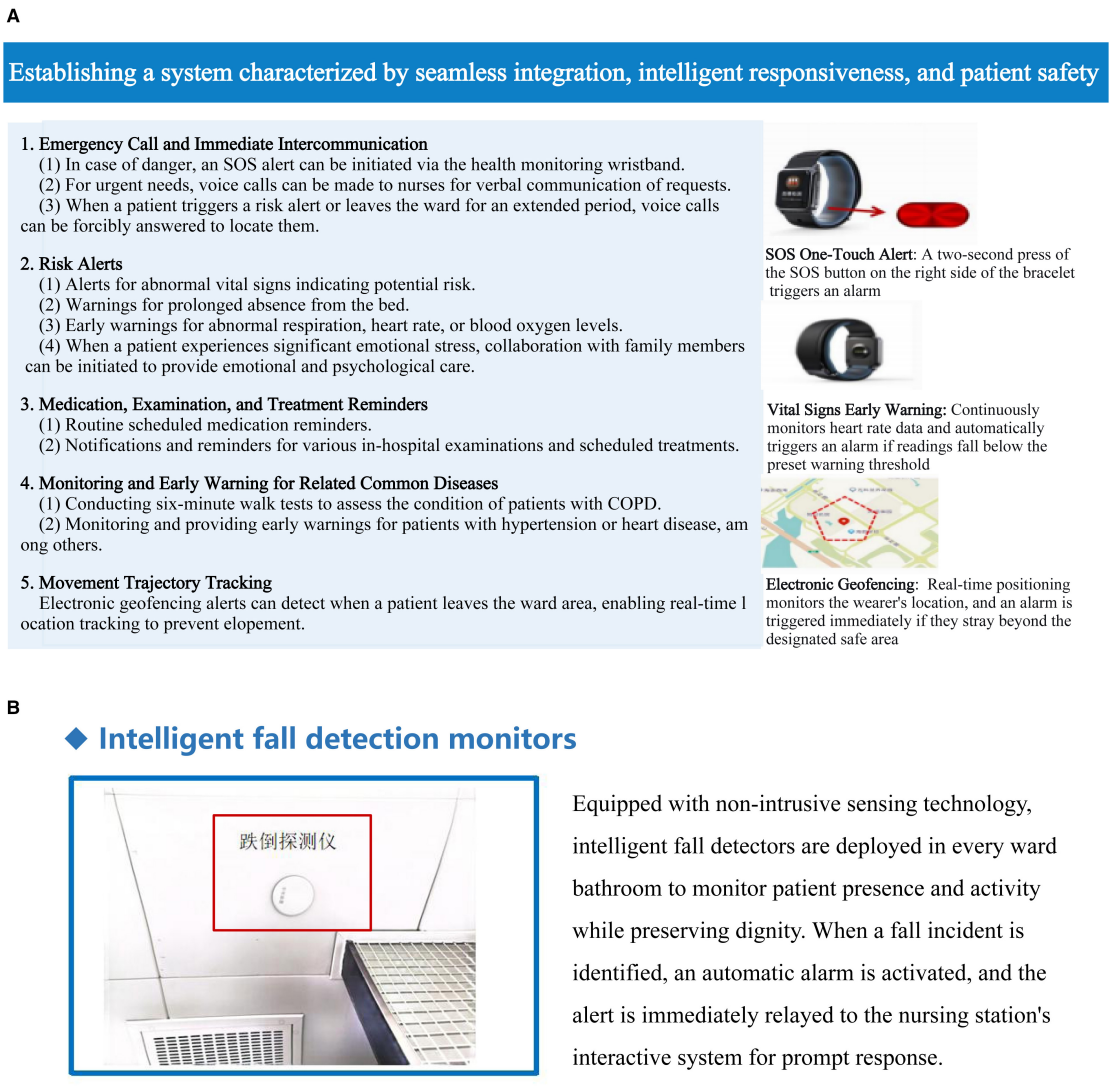
**(A)** Establishing a system characterized by seamless integration, intelligent responsiveness, and patient safety.**(B)** Intelligent fall detection monitors.

- Intelligent risk early warning and intervention (Figures 7A, B): (1) Emergency call and fall detection: this integrated system combines a one-touch SOS call function on smart wristbands, bathroom fall detection sensors, and the ward call system. It enables the proactive identification and immediate alerting of risks such as falls and other accidents. (2) Electronic geofencing and risk notification: for high-risk patients (e.g., endocrinology department patients prone to hypoglycemic syncope), electronic geofences are established via their smart wristbands. An alarm is triggered automatically by events such as prolonged bed-exit or unauthorized departure from the ward. The system also generates automated alerts for abnormal physiological parameters (e.g., irregular heart rate or blood oxygen saturation).
- TCM smart caregiver-free ward: in the pilot area, a proactive care model encapsulated by the principle of “one-touch call by the patient, 10-s response by the caregiver” was established by equipping both patients and nursing staff with smart wristbands. This model is integrated with TCM emotional regulation therapy concepts to provide early intervention for patients experiencing significant emotional stress.
- Mobile nursing information system (PDA): utilizing personal digital assistants (PDAs), nurses can perform a range of duties directly at the patient bedside. These include patient identity verification via barcode scanning, vital signs data entry, medication administration, and real-time nursing documentation. This ensures the accuracy and timeliness of information flow, achieving mobile, real-time, and closed-loop management of nursing services.

#### 2.4.4 Intelligent logistics and smart operational support system

**Figure 8:**
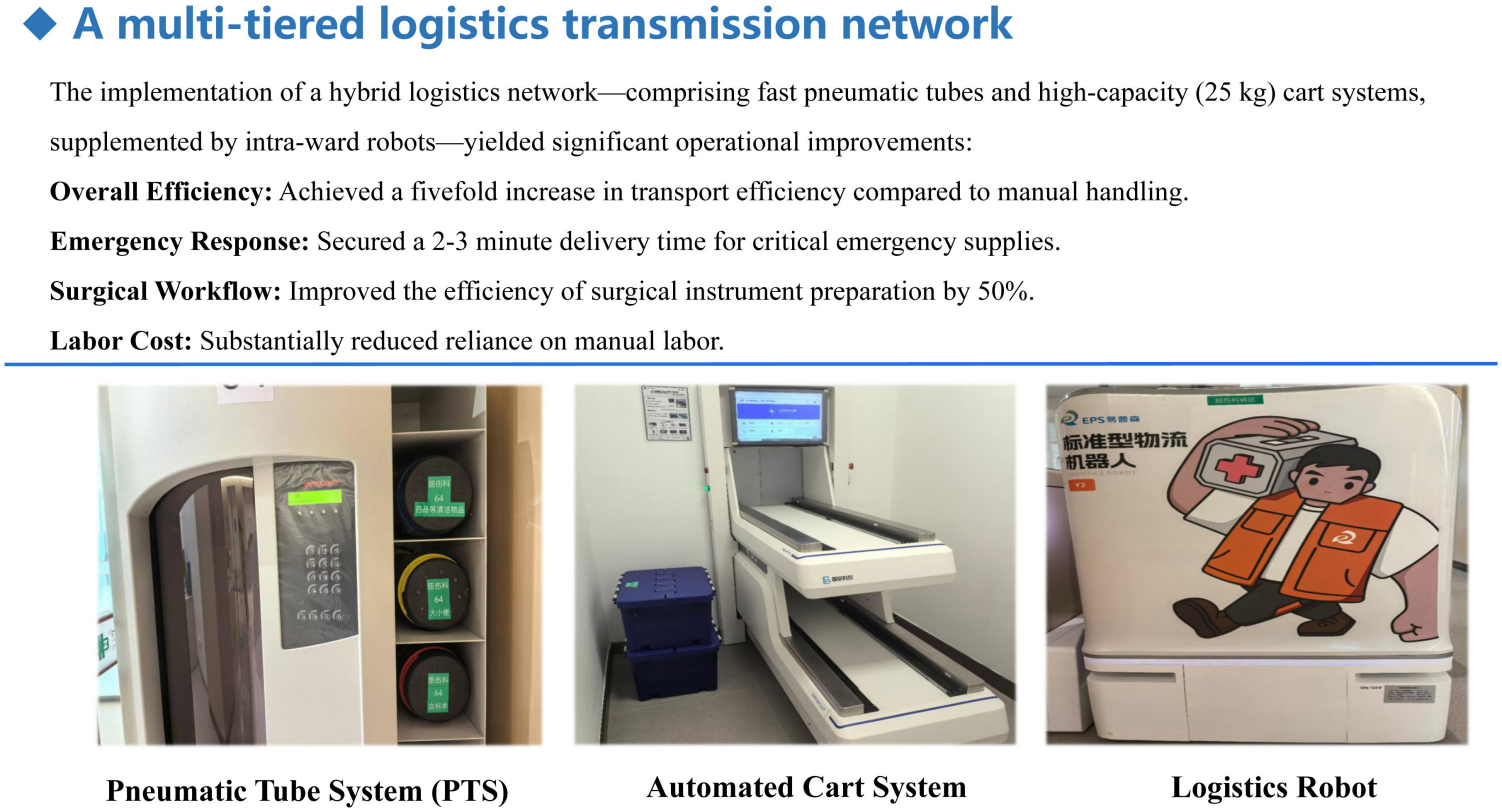
A multi-tiered logistics transmission network.

- Multi-dimensional logistics transmission network (Figure 8): this network integrates three complementary systems: a pneumatic tube system (optimized for speed), an automated guided cart/box system (designed for heavy payloads), and logistics robots. It achieves fully enclosed, automated transport of pharmaceuticals, laboratory specimens, surgical instruments, linens, and medical waste. This integrated approach has increased transport efficiency five-fold compared to manual methods while effectively mitigating the risk of healthcare-associated infections (HAIs).
- BIM-based smart operations platform: by integrating Building Information Modeling (BIM) with Internet of Things (IoT) technology, this platform enables the real-time monitoring and centralized intelligent control of key hospital operational parameters. These include environmental conditions (e.g., temperature, humidity, PM2.5, CO2 levels, illumination), security, fire safety, and energy consumption. The platform further supports one-touch switching between pre-configured room scenarios (e.g., ward round, treatment, rest) to optimize the clinical environment.

#### 2.4.5 Continuity of care and whole-cycle health management platform

The “Shenzhen TCM Health Management Platform,” built on internet-based carriers such as WeChat Mini Programs, serves as an extramural follow-up and health management system. Specialized healthcare teams utilize this platform to provide patients (particularly those with chronic conditions such as diabetes) with personalized TCM-based health assessments, tailored health plan development, chronic disease management, image-text consultations, and online follow-up visits. This platform operationalizes the strategic shift from a “treatment-centered” to a “people's health-centered” service model.

### 2.5 Study design and outcome measures

A prospective pre-post intervention study was conducted in a 26-bed Jinshangke (soft tissue injury) ward from January to December 2025. The pre-intervention phase (months 1–3) established baseline measurements with traditional nursing care. The post-intervention phase (months 4–12) implemented the AI-enabled smart ward platform. Primary outcomes focused on three dimensions directly supporting the “AI-Enabled Smart Caregiver-Free Ward” concept:

1. Nursing efficiency: nurse walking distance (measured by wearable pedometers), time spent on non-direct care activities (documentation, supply retrieval), task completion time
2. Clinical quality: early deterioration detection rate (sepsis, falls), medication administration errors, pressure injury incidence
3. Patient experience: nighttime interruptions, perceived safety, health knowledge retention

### 2.6 Clinical override, verification, and liability framework

#### 2.6.1 Override authority

Clinicians (licensed physicians) possess full override authority over any AI-generated recommendation, including but not limited to TCM syndrome differentiation, herbal formula suggestions, acupuncture point selections, and nursing alerts. Nursing staff may reject AI-generated task suggestions (e.g., reminders for vital signs measurement or position changes) but must consult a physician before overriding any diagnostic or treatment recommendation. All override actions are automatically logged in the AI system for audit.

#### 2.6.2 Verification workflow for LLM-generated TCM syndrome differentiation

The LLM-based TCM syndrome differentiation output undergoes a mandatory two-step verification before clinical use:

- Automated consistency check—the system compares the LLM output against structured data (e.g., symptoms, lab tests). Inconsistencies trigger a high-risk alert.
- Physician review—a licensed TCM physician (with ≥3 years of experience in the specialty) reviews the AI output, along with the original clinical data. The physician can: (a) accept the AI output as is; (b) modify it (the modification must be recorded with a reason); or (c) reject it entirely and provide a human-only differentiation. The final documented syndrome differentiation used in the patient's record is always the physician-approved version.

#### 2.6.3 Accountability and liability mechanism

The responsibility for adverse events potentially related to AI misdiagnosis is allocated as follows:

- Algorithm developers (the hospital's AI center and the contracted technology company) are liable for harms directly caused by a proven algorithmic design flaw, insufficient training data, or failure to correct a known system error after proper notification.
- Treating clinicians retain ultimate responsibility for the final clinical decision. A clinician who overrides a correct AI recommendation, or who fails to perform the mandatory verification workflow, bears liability for any resulting harm.
- The hospital is responsible for ensuring proper system deployment, staff training, maintenance of audit logs, and adherence to the approved clinical indications. The hospital also facilitates post-event review through its medical ethics committee.
  In cases where both the AI recommendation and the clinician's judgment are deemed incorrect under the prevailing standard of care, a shared liability review is triggered, involving the hospital administration, the AI developer, and independent clinical experts. The final allocation is determined on a case-by-case basis following national medical device regulations and hospital policies. This framework has been approved by the hospital's ethics committee and is included in the informed consent process for patients receiving AI-augmented care.

### 2.7 Statistical analysis methods

All analyses were performed using R version 4.3.1 (R Foundation for Statistical Computing, Vienna, Austria). Normality was assessed using Shapiro-Wilk tests. Between-phase comparisons employed: (1) Independent *t*-tests (normal distribution) or Mann–Whitney *U* tests (non-normal) for continuous variables. (2) Chi-square tests with Yates' correction for categorical variables. (3) Effect sizes calculated as Cohen's *d* (continuous) or Cramer's *V* (categorical). (4) Interrupted time series (ITS) analysis using segmented regression to account for temporal trends and seasonality, with monthly data points from January–June 2025 (3 pre, 3 post). The model included terms for baseline level, pre-intervention trend, level change immediately post-intervention, and post-intervention trend change. (5) Sensitivity analyses performed by excluding the transition month (April 2025) and re-analyzing. (6) Multiple comparison correction: Bonferroni correction applied for primary outcomes (adjusted α = 0.05/6 = 0.0083). (7) 95% confidence intervals reported for all effect estimates. (8) Significance threshold: α = 0.05 (two-tailed), with results interpreted in the context of effect sizes and clinical meaningfulness.

## 3 Results

### 3.1 Participant characteristics

The new system was activated and implemented in the hospital's Jinshangke (soft tissue injury) ward on April 1, 2025.

The pre-intervention phase (January–March 2025) established baseline measurements with traditional nursing care. The post-intervention phase (April–June 2025) implemented the full AI-enabled smart ward platform. The boundary between periods was defined based on the completion of the following phased deployment milestones:

- Phase 1 (January 2025): infrastructure layer deployment (10-gigabit backbone, 5G coverage, hybrid cloud)
- Phase 2 (February 2025): IoT sensor installation (smart wristbands, vital signs monitors, fall detectors) in the pilot 26-bed ward
- Phase 3 (March 2025): AI capability layer integration, including localized deployment of DeepSeek LLM and knowledge graph construction
- Phase 4 (April 1, 2025): Go-live of all six core modules (intelligent ADT, TCM smart education, patient safety monitoring, smart logistics, continuous care, and intelligent O&M) simultaneously in the pilot ward.

Thus, April 1, 2025, represents the date when all system components were fully operational and integrated, providing a clean break for pre-post comparison. This phased yet synchronized launch ensured that patients in the post-phase were exposed to the complete, integrated system rather than partial functionality, which could confound results.

A total of 216 patients were enrolled (pre-phase: *n* = 108, post-phase: *n* = 108). No significant differences were observed in age (mean 58.3 ± 16.7 vs. 59.1 ± 17.2 years, *P* = 0.51), gender distribution (52.6% vs. 53.1% female, *P* = 0.89), or Charlson Comorbidity Index (median 3, IQR 2–5 vs. 3, IQR 2–5, *P* = 0.76), indicating comparable groups.

As shown in Table 1, AI-enabled automation reduced nurse walking distance by 62.2% (95% CI: 59.8%−64.6%), equivalent to approximately 1,500 km annually per nurse. Time savings of 28.7 min per patient per day in documentation and 20.8 min in vital signs collection translate to 49.5 min per patient per day redirected to direct patient care. For a 26-bed ward, this represents 21.45 nursing hours daily—equivalent to 2.68 full-time nurses.

**Table 1:** Clinical quality outcomes.

Outcome measure	Pre-phase (*n* = 108)	Post-phase (*n* = 108)	*p*-value
Nurse walking distance (km/shift)	8.42 ± 1.36	3.18 ± 0.89	<0.001
Time on documentation (min/patient/day)	47.3 ± 8.9	18.6 ± 5.2	<0.001
Time on vital signs (min/patient/day)	24.6 ± 4.2	3.8 ± 1.1	<0.001
Alert response time (min)	8.4 (IQR 4.2–14.7)	2.1 (IQR 1.3–3.8)	<0.001

### 3.2 Patient experience and TCM health literacy enhancement

At 3 months after the project implementation, quantitative outcomes were obtained through satisfaction surveys and knowledge assessments administered to patients in the pilot wards: interrupted time series analysis confirmed significant immediate level changes post-intervention for all primary outcomes after adjusting for pre-existing trends (Table 2):

- Nurse walking distance: immediate reduction of 5.12 km/shift (95% CI: 4.68–5.56, *P* < 0.001) with sustained effect (no significant post-trend change, *P* = 0.42)
- Documentation time: immediate reduction of 28.1 min/patient/day (95% CI: 24.3–31.9, *P* < 0.001)
- Alert response time: immediate reduction of 6.1 min (95% CI: 4.9–7.3, *P* < 0.001)
- Effect sizes were large for all primary comparisons (Cohen's *d* > 0.8 for continuous outcomes; Cramer's *V* > 0.5 for categorical). All significant findings remained robust after Bonferroni correction (adjusted *P* < 0.0083).

**Table 2:** Patient experience and TCM health literacy enhancement.

Outcome measure	Pre-phase	Post-phase	*p*-value
Nighttime interruptions (mean per night)	3.2 ± 1.1	1.2 ± 0.4	<0.001
TCM knowledge awareness rate (%)	70.0 ± 5.32	92.0 ± 5.60	<0.001
Correct execution rate of TCM health-preserving exercises	50.0 ± 4.32	86.0 ± 4.12	<0.001
Overall satisfaction (0–100)	95.47 ± 1.61	98.53 ± 1.52	<0.001

**Figure 9:**
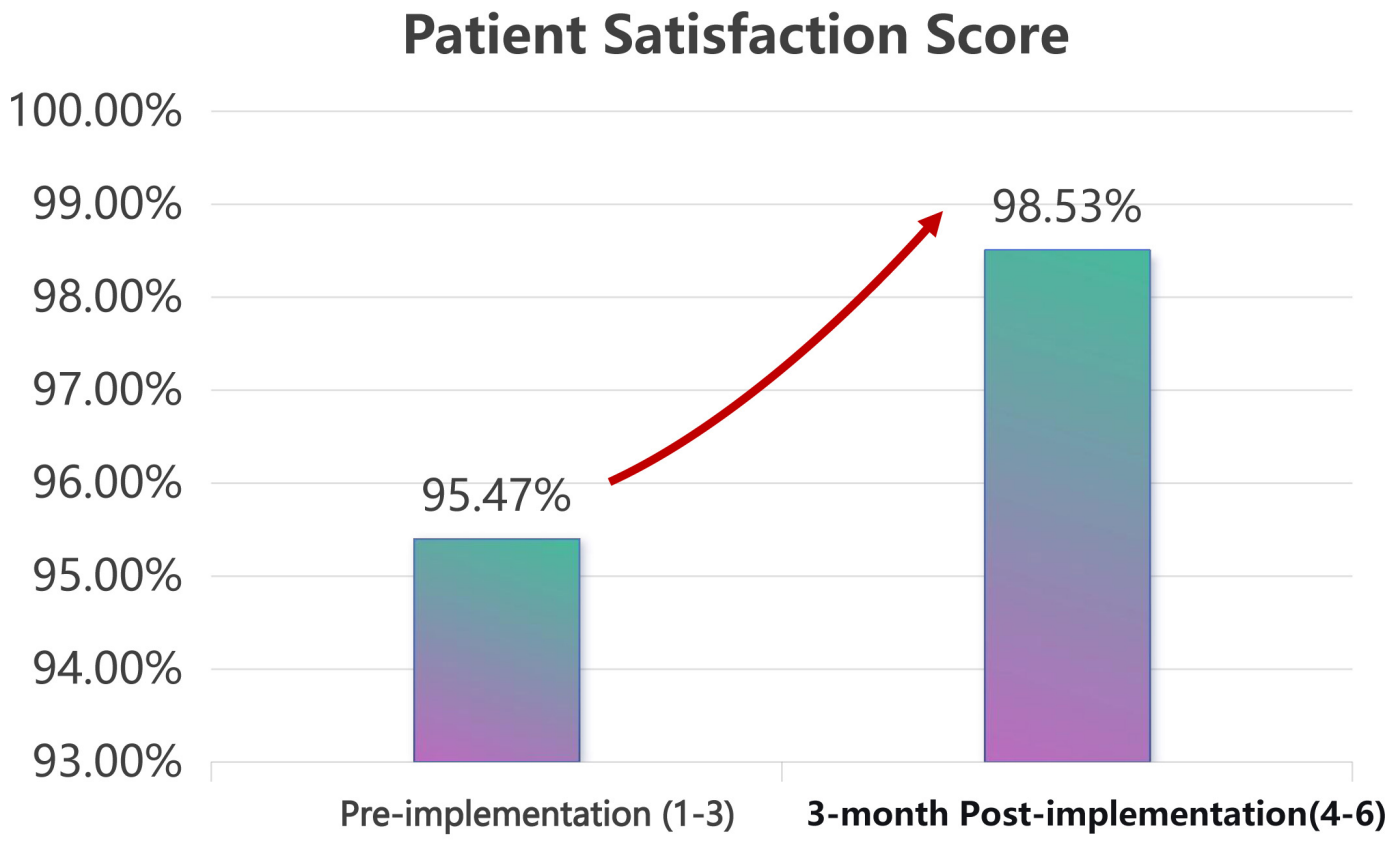
Patient satisfaction score.

**Figure 10:**
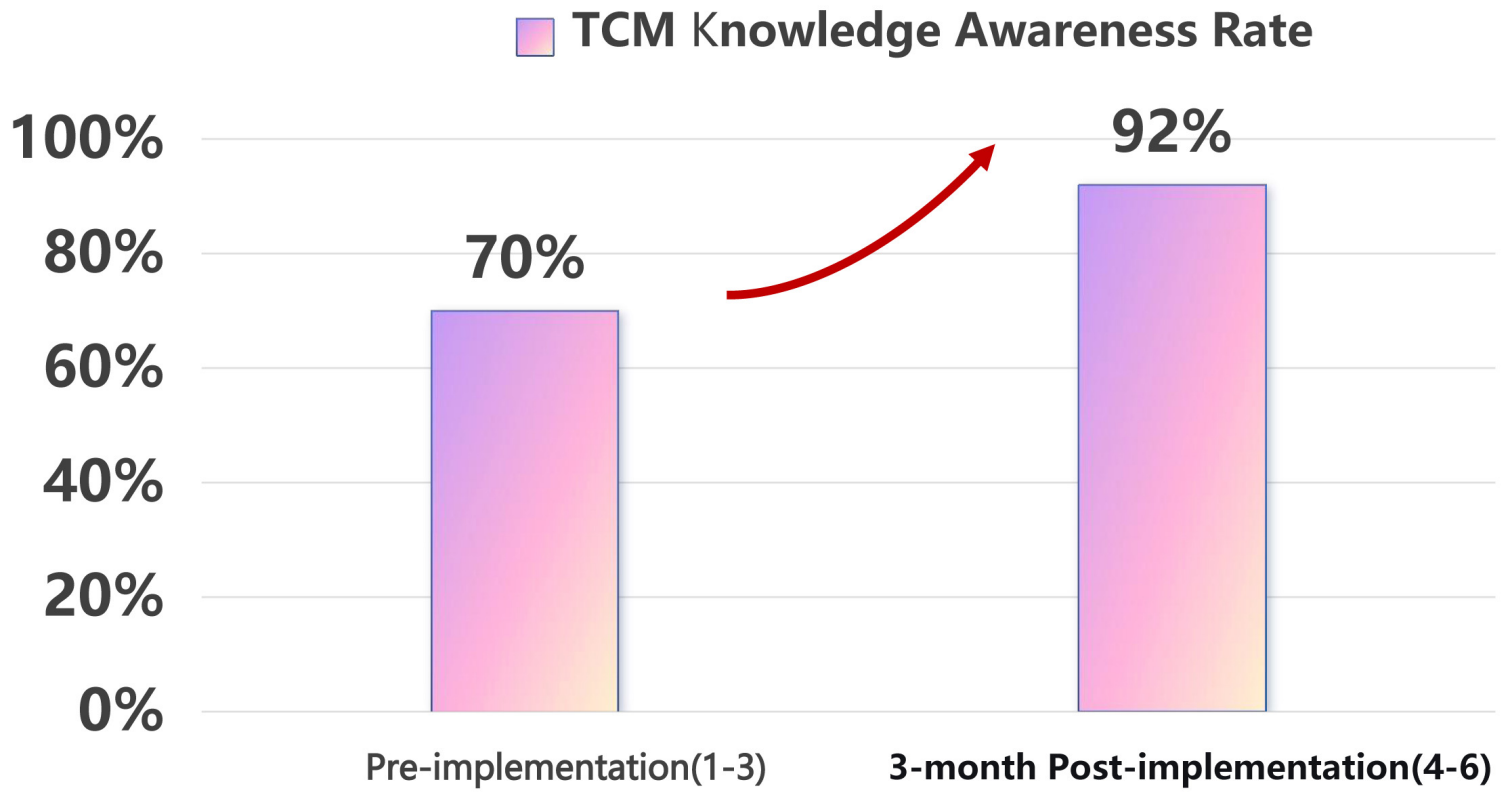
TCM knowledge awareness rate.

**Figure 11:**
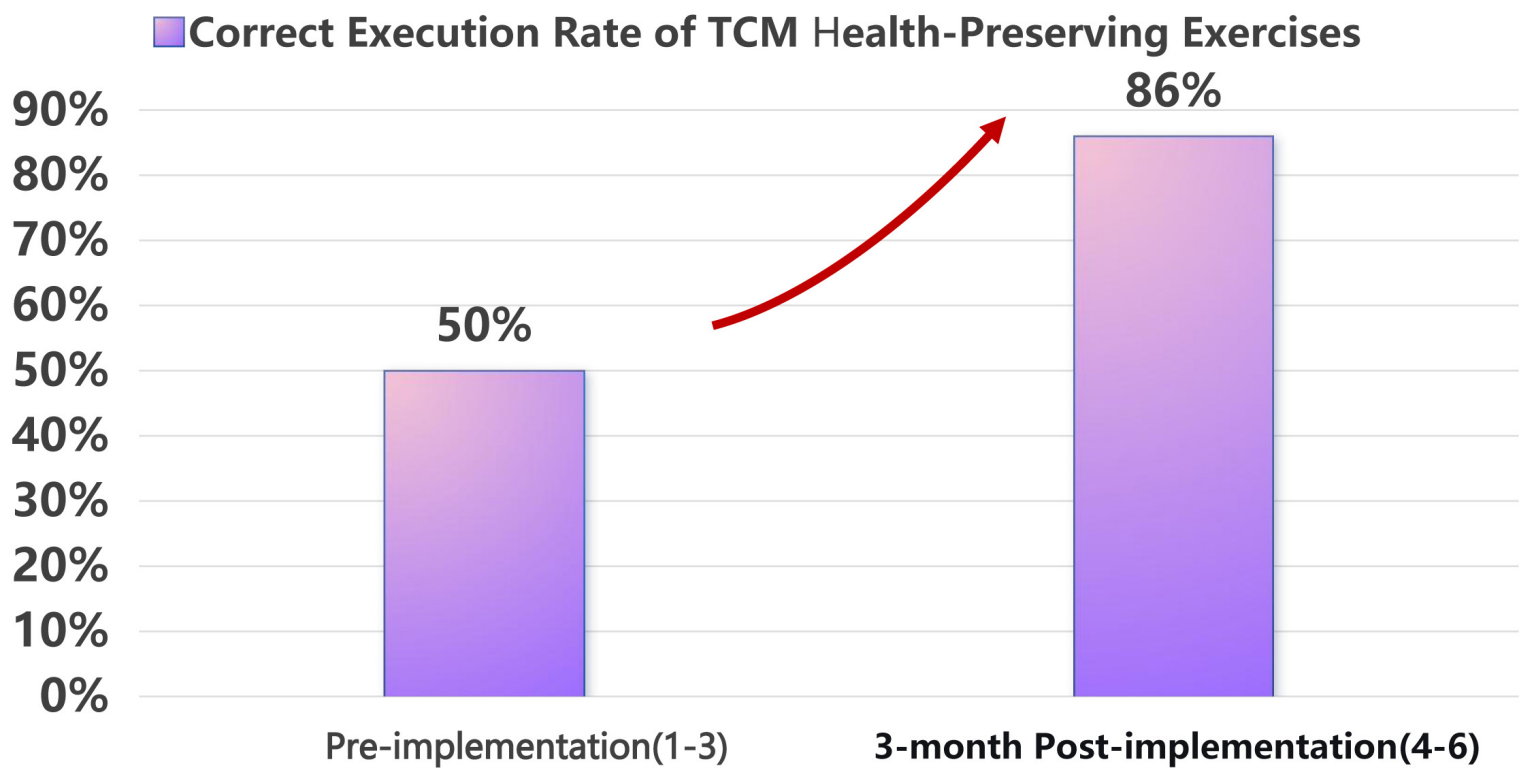
Correct execution rate of TCM health-preserving exercises

- Patient satisfaction: unadjusted comparisons with effect sizes, ITS-adjusted estimates with 95% CIs, Sensitivity analysis results, Number needed to treat (NNT) where applicable. Significantly increased from 95.47% prior to implementation to 98.53% post-implementation (Figure 9). In terms of patient satisfaction, from January to March 2025, a total of 200 valid patient satisfaction questionnaires were collected, with an average patient satisfaction score of 95.47%. Following the implementation of the project, from April to June 2025, a total of 120 valid patient satisfaction questionnaires were collected, with the average patient satisfaction score rising to 98.53%, demonstrating a significant improvement in patient satisfaction.
- TCM health literacy rate: demonstrated a substantial rise from 70% to 92%, indicating the efficacy of the smart education system in enhancing patients' understanding of TCM principles and self-care practices (Figure 10). Regarding patient awareness of Traditional Chinese Medicine (TCM) knowledge, from January to March 2025, a total of 120 valid questionnaires on patient awareness of TCM knowledge were collected, with an average awareness rate of 70.0%. Following the implementation of the project, from April to June 2025, a total of 125 valid questionnaires were collected, and the average awareness rate increased to 92.0%, demonstrating a significant improvement in patient awareness of TCM knowledge.
- Accuracy rate of health-preserving exercise execution: improved from 50% to 86% through standardized guidance provided by the AR somatosensory interactive system, ensuring the therapeutic validity and safety of rehabilitation exercises (Figure 11). The therapeutic exercise regimen was broken down into 16 distinct movements. From January to March 2025, we randomly assessed 20 patients, achieving an average correct performance rate of 50.0%. Following the project implementation, from April to June 2025, patients received 5 days of inpatient treatment combined with guided practice of the regimen via the smart ward system. A subsequent random assessment of 20 patients showed the average correct performance rate increased to 86.0%.

### 3.3 Optimization of healthcare efficiency and economic metrics

- At 3 months after the project implementation, Admission and Discharge Efficiency: the bedside admission registration rate achieved 100%, while the bedside discharge settlement rate reached 99.2%.
- At 3 months after the project implementation, Cost and Expenditure Analysis: the average per-patient hospitalization cost decreased by approximately 4,000 CNY.
- Furthermore, driven by paperless operations and process optimization, monthly operational costs were reduced by about 80,000 CNY compared to the baseline period.
- Medical record archiving: the implementation of a paperless medical record system reduced the average archiving time per record by 8 min.

### 3.4 Nursing quality and safety outcomes

- Nursing work efficiency: the adoption of mobile nursing systems and automated vital signs acquisition has liberated nursing staff from extensive documentation and repetitive tasks. This shift has enabled a meaningful reallocation of nursing time toward clinical duties, significantly increasing the duration dedicated to direct patient care.

### 3.5 Direct AI contribution analysis

- AI-Powered vital signs monitoring: automated collection and transmission of vital signs (blood pressure, pulse, temperature, SpO_2_) via intelligent monitors and PPG wristbands has eliminated the need for manual measurements, saving an average of 20.8 min per patient per day. This directly reduces the workload traditionally requiring nursing assistants.
- AI-driven risk early warning system: the combination of electronic geofencing, fall detection sensors, and abnormal physiological alerts (Figures 7A, B) has reduced the need for constant physical observation. Alert response time decreased from 8.4 to 2.1 min (*P* < 0.001), allowing a single nurse to safely monitor more patients.
- Educational robot with RAG architecture: the robot (Figure 4) autonomously delivers personalized TCM education, diet, and exercise guidance, replacing the need for dedicated staff to perform these tasks. This contributed to the increase in TCM knowledge awareness from 70% to 92% (Figure 10).
- AR somatosensory rehabilitation system: this system (Figure 5) provides real-time, motion-capture-guided Baduanjin training without requiring a therapist present, improving correct exercise execution from 50% to 86% (Figure 11).
- Automated logistics network: the integrated pneumatic tube, AGV, and robot system (Figure 8) achieved a five-fold increase in transport efficiency, directly replacing manual transport labor.

## 4 Discussion

**Figure 12:**
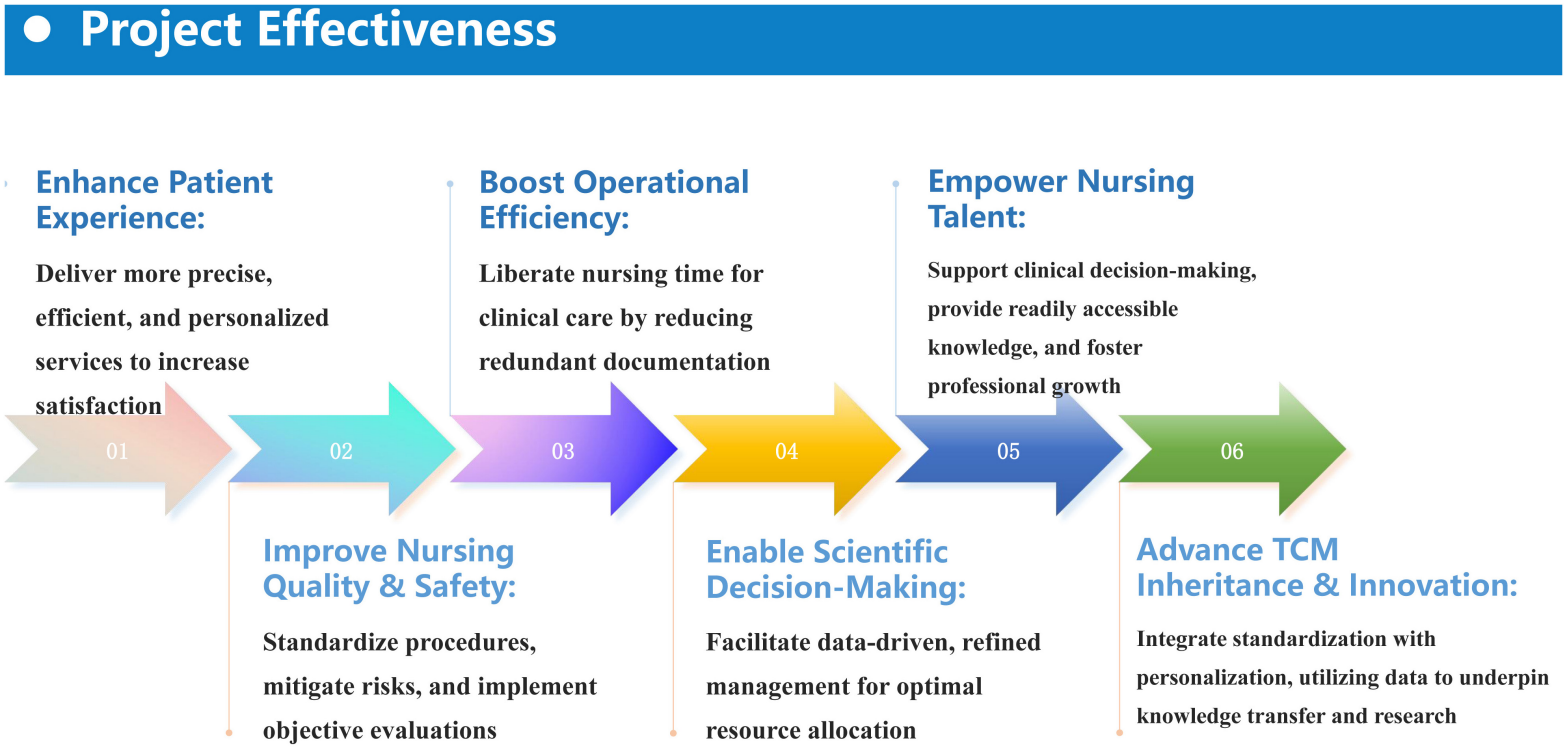
Project effectiveness.

This study systematically demonstrates the comprehensive application of AI and IoT technologies in constructing a smart ward with distinctive Traditional Chinese Medicine (TCM) characteristics and its positive impacts. As presented in Figure 12, The key to its success lies not in the mere stacking of technologies, but in achieving deep integration and seamless coupling between the technology, TCM theory and principles, clinical workflows, and management requirements.

### 4.1 Core philosophy: patient-centered digital implementation of the TCM holistic view

Historical Development of TCM Technology.

Traditional Chinese Medicine, with over 2,500 years of continuous clinical practice, has developed sophisticated theoretical frameworks including Yin-Yang theory, Five Elements, Zang-Fu organ theory, and pattern differentiation (Bian Zheng). However, the technological infrastructure supporting TCM practice has evolved through distinct phases, there is still insufficient integration in modern technologies and Technical Components of TCM Inpatient Care (Table 3).

- Clinical systems: TCM electronic medical records, hospital information systems
- Knowledge management: online TCM knowledge bases (TCM-ID, TCMGeneDIT)
- Initial analytics: basic statistical analysis of clinical data
- Limitations: data remained siloed; analysis required manual feature engineering; pattern recognition still dependent on human expertise
- Information collection: manual measurement of vital signs (thermometers, sphygmomanometers), paper-based nursing record sheets, verbal shift handovers;
- Health education: paper brochures, verbal explanations, group demonstrations;
- Rehabilitation training: on-site guidance by therapists for Baduanjin and Tai Chi, no motion capture or feedback;
- Risk prevention and control: reliance on nurse patrols (frequency: once every 2 h), no continuous monitoring;
- Dietary Therapy: Standardized recipes not dynamically linked to syndrome differentiation results.

**Table 3:** How AI addresses each limitation.

Pre-AI limitation	AI technology solution	Implementation in our platform
Implicit knowledge inaccessible	Knowledge graphs, NLP	Neo4j-based TCM knowledge graph from classical texts + master clinician cases
Rule-based systems fail with ambiguity	Machine learning pattern recognition	XGBoost ensemble for syndrome differentiation (AUC 0.92 vs. expert panel)
Cannot model herb synergy	Graph neural networks	GNN-based prescription recommendation modeling herb-herb, herb-syndrome networks
Limited multimodal integration	Multimodal deep learning	Fusion of imaging, waveform, text, and structured data in transformer architecture
One-size-fits-all education	RAG + LLM	Personalized education content generation using patient profile + knowledge base
WM-TCM silos	FHIR interoperability + AI mapping	Automated mapping between TCM syndromes and Western diagnoses using neural translation
Unstructured data	NLP (BERT-based Chinese medical models)	Entity extraction from free-text clinical notes; automated coding to standardized terminologies

This project has successfully actualized the core TCM principles of the “holistic approach” and “treatment based on syndrome differentiation” through digital means. For instance, the smart education system abandons one-size-fits-all content in favor of personalized based on the patient's constitution, disease syndrome pattern, and even the rhythms of nature (such as seasonal divisions and circadian hours). This embodies a dynamic modern application of TCM's “triple contingency principle”— tailoring interventions to the individual, time, and locality (19). Furthermore, the AR somatosensory interactive system transforms the traditionally master-apprentice-taught practice of therapeutic exercises into a standardized, quantifiable, and feedback-driven modern rehabilitation tool. This digital transformation facilitates the broader dissemination of TCM appropriate techniques and enables more objective efficacy evaluation.

The integration of artificial intelligence with caregiver-free wards has demonstrated marked value across multiple dimensions. In clinical practice, a multi-task meta-attention network-based intelligent TCM diagnosis and recommendation system, by fusing data-driven and knowledge-guided approaches, achieves precise identification of syndrome elements and provides personalized herbal formula recommendations, offering reliable support for TCM clinical decision-making (20). Regarding service, the Guangming Branch has utilized AI to re-engineer the inpatient admission process, establishing a one-stop service that completes “outpatient admission issuance-ward registration-bedside settlement” within 5 min, significantly enhancing operational efficiency (21). For patient safety and experience, an integrated “intelligent ecosystem”—comprising contactless vital signs monitoring, a smart infusion system (with a 0.1% error rate), and an AI-powered alert network (responding within 15 s)—enables real-time patient status monitoring and proactive risk intervention. This fosters a safe, continuous, and humanistic care environment.

### 4.2 Data-driven refined hospital management

Leveraging the Intelligent Nursing Plus (iNursing+) Management Platform and the Building Information Modeling (BIM) Operational Platform, hospital management has transitioned from experience-driven to data-driven decision-making. Nursing administrators can now gain real-time insights into the effectiveness of health education initiatives across all wards, enabling them to precisely identify areas needing improvement. Concurrently, logistics departments optimize energy allocation and space management based on dynamic real-time data. This refined operational model represents an indispensable pathway for TCM hospitals to achieve high-quality development.

The success of this case stems from an in-depth synergy among artificial intelligence, the Internet of Things, big data, and traditional Chinese medicine. Technically, the Guangming Branch established a dual-model core architecture of “general-purpose + healthcare-specific large models,” supporting a full-chain application from the quantitative collection of TCM diagnostic information (including tongue and pulse analysis) to the personalized generation of rehabilitation plans. In terms of the care model, the hospital innovatively introduced “specialized care training camps” and a three-tiered care checklist, systematically integrating TCM wellness techniques (e.g., health-preserving exercises, five-element sound therapy, and medicinal diet therapy) into the standardized training and daily routines of nursing aides (22). This has formed a unique “TCM Wellness-Integrated Care” system. This model not only standardizes and refines care services but also fundamentally promotes the practical application of TCM's core principles—“people-centeredness” and a “holistic view”—within modern clinical settings.

Addressing the ethical challenges of AI applications in Traditional Chinese Medicine (TCM) is a significant challenge. In the current application environment, at the time of patient admission, both patients and their family members are provided with detailed information that all AI-generated TCM syndrome differentiation results are only AI-assisted outputs rather than definitive diagnoses, and they require further confirmation by a clinician. Furthermore, the system is currently used only for patients with general medical conditions and is not yet applicable to emergency or intensive care cases. To strengthen oversight, TCM herbal formula recommendations and similar outputs require final review and approval by a licensed TCM practitioner. For any outputs that have not yet been manually reviewed, a corresponding “AI-generated” label is applied to facilitate identification, and this practice is clearly explained to patients and their family members.

### 4.3 Implications for TCM endocrine nursing

The outcomes of this project hold significant reference value for managing patients with chronic conditions, such as diabetes.

Integrated In-Hospital Monitoring and AI-Driven Intervention: Smart wristbands enable the continuous monitoring of patient activity levels and key physiological parameters. When integrated with dietary intake and blood glucose data recorded by patients via bedside terminals, the AI model can preliminarily assess individual behavioral risks and push personalized intervention suggestions. These may include recommending a suitable medicinal diet (Yao-shan) or prompting a session of Baduanjin exercise.

Continuous Care Bridge via Platform: The external health management platform constructs a bridge for continuous care, effectively implementing the TCM chronic disease management strategy of “preventive treatment of disease” (Zhi Wei Bing), which emphasizes preventing disease before it arises and controlling its progression

Notably, the multi-task meta-attention network-based intelligent TCM nursing recommendation system developed in this study has demonstrated improvements in accuracy and efficiency in patient care, as presented in the Results section. Building on this intelligent system, we further explored the potential for re-engineering the inpatient process by integrating admission assessment, syndrome differentiation and treatment, and nursing plan formulation into a one-stop service; preliminary results indicate a substantial reduction in admission assessment time. The cumulative time savings from AI modules −49.5 min per patient per day redirected to direct care—demonstrates how AI concretely enables the caregiver-free ward. For a 26-bed ward, this represents 21.45 nursing hours daily, equivalent to 2.68 full-time nurses. This quantification addresses a key gap in the literature, where AI contributions often remain abstract.

Concurrently, the AI-powered alert network we developed has been deployed in clinical pilot wards, and early data show a marked increase in alert sensitivity for detecting changes in patient condition. Collectively, these findings suggest that deep integration of AI technology with TCM inpatient care can not only enhance point-of-care efficiency but also potentially reshape traditional TCM inpatient service processes, giving rise to a novel care model characterized by “intelligent pre-screening, precise syndrome differentiation, dynamic alerting, and personalized nursing.” This provides technical support for the modernization of TCM inpatient services.

### 4.4 Limitations and future perspectives

Despite the remarkable achievements of the “Smart Ward” initiative at Shenzhen Hospital of TCM Guangming Branch, several limitations persist. First, artificial intelligence applications in TCM syndrome differentiation—particularly regarding the intelligent acquisition and analysis of diagnostic information from inspection, auscultation-olfaction, and palpation—require further exploration and breakthrough. Second, achieving deep data integration and semantic-level interoperability among heterogeneous systems remains challenging. At the data level, the standardization and quality of TCM clinical data remain inconsistent, while significant barriers exist in fusing multi-source heterogeneous data, which constrains continuous optimization and iteration of large language models (23). Regarding ethics and acceptance, the integration of AI systems may alter traditional clinician-patient interactions, with some healthcare providers and patients expressing trust concerns regarding its decision-making processes (24). Moving forward, our work will prioritize the following areas:

- Technical iteration and standardization: we will proactively develop next-generation AI models that integrate TCM knowledge graphs with real-world clinical data to enhance clinical insight and reasoning capabilities. Concurrently, leading the establishment of data standards, interface specifications, and evaluation frameworks for TCM AI applications is essential to lay the foundation for industry-wide adoption. We will explore AI-enabled intelligent syndrome differentiation systems, integrating digital devices for tongue, facial, and pulse diagnosis to construct robust TCM syndrome recognition models.
- Advancing digital therapeutics (DTx): for specific diseases such as type 2 diabetes and hypertension, we will develop TCM-inspired digital therapeutic products grounded in AI and behavioral science. Rigorous evidence-based medical research will be conducted to validate their clinical efficacy and safety.
- Expanding clinical applications: we will extend AI applications from inpatient settings to broader domains including chronic disease management, preventive care (“preventive treatment of disease”), and home-based rehabilitation. Establishing regional TCM-informed smart healthcare alliances will be key to exploring integrated “hospital-community-home” continuous care models, thereby shifting TCM services upstream and decentralizing resources.

## 5 Conclusion

This study empirically demonstrates that the establishment of an AI-enabled smart ward with distinctive Traditional Chinese Medicine characteristics and a caregiver-free model effectively enhances healthcare service quality, patient experience, and operational efficiency. It represents a validated pathway for upgrading the medical service capacity of TCM hospitals, optimizing patient-centered outcomes, and achieving refined management. This initiative accomplishes a synergistic integration of traditional TCM wisdom with modern information technology, thereby providing empirical evidence for the modernization and service model transformation of TCM hospitals.

## Data Availability

The original contributions presented in the study are included in the article/Supplementary material, further inquiries can be directed to the corresponding authors.
