# Supplementary material for: Exploration and practice of AI-enabled smart caregiver-free ward with traditional Chinese medicine characteristics: a case study based on the Guangming branch of Shenzhen Traditional Chinese Medicine Hospital
Source: Front Public Health. 2026 Jun 2;14:1799247. doi: 10.3389/fpubh.2026.1799247 (PMC13269425; doi:10.3389/fpubh.2026.1799247)
Supplement: Supplementary file 1 [file Data_Sheet_1.docx]

| ****Module**** | ****Function**** | ****Input Data**** | ****Output**** | ****Integration**** |
| --- | --- | --- | --- | --- |
| ****Clinical Decision Support (CDS)**** | Sepsis prediction, deterioration alert | Vital signs, lab results (FHIR Observation) | Risk scores (0-100), alert levels | Publishes to Kafka topic *ai.alert.generated*; consumed by nurse dashboard |
| ****TCM Diagnostic Engine**** | Tongue/pulse analysis, syndrome differentiation | Tongue images (DICOM), pulse waveforms, symptom text | TCM syndrome type, confidence scores | FHIR *Observation* with extensions for TCM; stored in clinical data lake |
| ****Herbal Prescription Recommender**** | Formula generation based on syndrome | Syndrome type, patient history, contraindications | Recommended formula with dosage | FHIR *MedicationRequest;* integrated with pharmacy system |
| ****Educational Robot Controller**** | Personalized patient education (RAG-based LLM) | Patient profile, diagnosis, learning preferences | Customized education content, Q&A responses | FHIR *Communication* resource; push to bad/ patients’ App |
| ****Outcome Predictor**** | Length of stay, readmission risk | Multimodal patient data | Predictive analytics dashboard | Real-time via WebSocket to management console |

**Supplementary Table 1. Knowledge Layer (AI Capability Layer)**

***Layer 1: Perception Layer (Data Acquisition)***

Data Output: Raw sensor data (time-series), HL7 v2.x messages, DICOM images

Interface Protocols: BLE 5.0, MQTT, HL7 MLLP, DICOM DIMSE

Destination: IoT Gateway Layer (via edge processing nodes)

***Layer 2: Network and IoT Gateway Layer***

Data Output: Normalized FHIR resources, filtered time-series data

Interface Protocols: MQTT, HTTPS, WebSocket, FHIR RESTful API

Destination: Data Bus/API Gateway Layer

***Layer 3: Data Bus and API Gateway Layer***

Data Output: Routed messages to appropriate consumers, audit trails

Interface Protocols: RESTful API, Kafka topics, HL7 FHIR R4

Destination: Knowledge Layer, Application Layer, external systems

***Layer 4: Knowledge Layer (AI Capability Layer)***

Data Output: AI-generated insights, predictions, recommendations

Interface Protocols: Internal gRPC, Kafka, FHIR RESTful

Destination: Application Layer, Data Storage Layer

***Layer 5: Application Layer***

End-user applications for different stakeholders

| ****System**** | ****Integration Method**** | ****Data Exchanged**** | ****Clinical/Management Decisions Enabled**** |
| --- | --- | --- | --- |
| ****HIS (Hospital Information System)**** | HL7 v2 ADT messages → FHIR Patient/Encounter via MLLP gateway | Demographics, admissions, discharges, transfers | Automated patient context for AI; bed management optimization |
| ****LIS (Laboratory Information System)**** | HL7 ORU messages → FHIR DiagnosticReport/Observation | Lab results, microbiology | Sepsis early warning; medication adjustment recommendations |
| ****PACS (Picture Archiving)**** | DICOM C-STORE + FHIR ImagingStudy | Radiological images, reports | AI-assisted image interpretation; finding correlation with TCM tongue diagnosis |
| ****Pharmacy System**** | FHIR MedicationRequest/MedicationDispense | Prescriptions, dispensing records | Drug interaction checking; TCM-western medicine integration alerts |
| ****Wearable Device Platform**** | MQTT → FHIR Observation via IoT Gateway | Continuous vitals, activity data | Early deterioration detection; rehabilitation progress monitoring |
| ****EMR (Electronic Medical Record)**** | FHIR bidirectional sync | Full clinical record | Comprehensive AI context; longitudinal analysis |

**Supplementary Table 2. Integration with Existing Hospital Systems**

| ****Flow ID**** | ****Source Layer/Component**** | ****Target Layer/Component**** | ****Data Content**** | ****Interface Protocol**** | ****Data Format**** | ****Trigger Mechanism**** | ****FHIR Resource Mapping**** |
| --- | --- | --- | --- | --- | --- | --- | --- |
| ****F1**** | Perception: Wearable sensors | Gateway: Edge node | Heart rate, SpO2, BP, temperature | BLE 5.0/MQTT | JSON (time-series) | Continuous (1 Hz) | FHIR Observation (vital signs) |
| ****F2**** | Gateway: Edge node | Data Bus: Kafka cluster | Filtered vitals, normalized data | MQTT/HTTPS | Avro (schema-registry) | Event-driven (change detection) | FHIR Bundle of Observations |
| ****F3**** | HIS (legacy) | Gateway: HL7 adapter | ADT messages (admissions, transfers, discharges) | HL7 MLLP (TCP) | ER7 (pipe-delimited) | Real-time (on event) | FHIR Patient, Encounter |
| ****F4**** | LIS (legacy) | Gateway: HL7 adapter | Laboratory results | HL7 MLLP (TCP) | ER7/XML | Batch (every 15 min) | FHIR DiagnosticReport, Observation |
| ****F5**** | PACS (legacy) | Gateway: DICOM adapter | Medical images, structured reports | DICOM C-STORE | DICOM Part 10 | On completion | FHIR ImagingStudy, DocumentReference |
| ****F6**** | Data Bus: Kafka topic *patient.data.raw* | Knowledge: CDS module | Multimodal patient data (vitals + labs) | Kafka consumer | Avro | Asynchronous (subscribe) | FHIR resources parsed internally |
| ****F7**** | Knowledge: TCM Diagnostic Engine | Data Bus: Kafka topic *ai.tcm.result* | TCM syndrome, tongue classification, confidence | Kafka producer | JSON + FHIR extensions | On demand/event | FHIR Observation (TCM profile) |
| ****F8**** | Knowledge: Educational Robot | Application: Patient portal | Personalized education content | HTTPS + WebSocket | HTML/JSON | Push notification | FHIR Communication |
| ****F9**** | Application: Clinical dashboard | Data Bus: Kafka topic *clinician.action* | Care plan updates, medication orders | REST API | FHIR CarePlan, MedicationRequest | User action | FHIR (direct storage) |
| ****F10**** | Data Bus: All topics | Storage: Clinical data lake | Archived data for analytics | Kafka Connect + JDBC | Parquet (HDFS) | Continuous | All FHIR resources |

**Supplementary Table 3. Detailed Data Flow Specifications Across Architecture Layers**
